# Exhaustive TORCH Pathogen Diagnostics Corroborate Zika Virus Etiology of Congenital Malformations in Northeastern Brazil

**DOI:** 10.1128/mSphere.00278-18

**Published:** 2018-08-08

**Authors:** Andres Moreira-Soto, Renata Cabral, Celia Pedroso, Monika Eschbach-Bludau, Alexandra Rockstroh, Ludy Alexandra Vargas, Ignacio Postigo-Hidalgo, Estela Luz, Gilmara Souza Sampaio, Christian Drosten, Eduardo Martins Netto, Thomas Jaenisch, Sebastian Ulbert, Manoel Sarno, Carlos Brites, Jan Felix Drexler

**Affiliations:** a Institute of Virology, University of Bonn Medical Centre, Bonn, Germany; b Charité-Universitätsmedizin Berlin, corporate member of Freie Universität Berlin, Humboldt-Universität zu Berlin, and Berlin Institute of Health, Institute of Virology, Berlin, Germany; c Maternidade Climério de Oliveira, Universidade Federal da Bahia, Salvador, Brazil; d Complexo Hospitalar Universitário Professor Edgard Santos, Universidade Federal de Bahia, Salvador, Brazil; e Department of Immunology, Fraunhofer Institute for Cell Therapy and Immunology, Leipzig, Germany; f German Centre for Infection Research (DZIF)‡; g Department for Infectious Diseases (Section Clinical Tropical Medicine), Heidelberg University Hospital, Heidelberg, Germany; University of Chicago

**Keywords:** Brazil, TORCH, Zika virus, microcephaly, parturient

## Abstract

The Latin American 2015–2016 Zika virus (ZIKV) outbreak was associated with an increase in microcephaly predominantly in northeastern Brazil. To comparatively investigate infectious causes of congenital malformations, we performed a nested case-control study in 32 mothers of cases of suspected congenital Zika syndrome (CZS) and 160 age-matched controls from Bahia, northeastern Brazil. We collected clinical and imaging data and assessed past exposure to ZIKV, Chikungunya virus (CHIKV), dengue virus, and 8 established TORCH (Toxoplasma gondii, Treponema pallidum, rubella virus, cytomegalovirus, herpes simplex virus 1 [HSV-1] and HSV-2, varicella-zoster virus, parvovirus B19) pathogens using multiple serological tests. Heterogeneous symptoms prevented unequivocal diagnosis of CZS on clinical grounds. Only ZIKV and CHIKV seroprevalence rates differed significantly between cases and controls (93.8% versus 67.8% for ZIKV [Fisher’s exact text, *P* = 0.002] and 20.7% versus 8.2% for CHIKV [χ^2^, *P* = 0.039]). High ZIKV seroprevalence rates in cases could not be explained by previous dengue virus infections potentially eliciting cross-reactive antibody responses affecting ZIKV serological tests. In conditional logistic regression analyses, only ZIKV was significantly associated with congenital malformations (*P* = 0.030; odds ratio, 4.0 [95% confidence interval, 1.1 to 14.1]). Our data support an association between maternal ZIKV exposure and congenital malformations. Parallels between the discrepant ZIKV and CHIKV seroprevalence rates between cases and controls and similar seroprevalence rates between cases and controls for the sexually transmitted T. pallidum and HSV-2 may suggest the occurrence of predominantly vector-borne transmission in our study population. High seroprevalence of TORCH pathogens suggests that exhaustive diagnostics will be necessary in the aftermath of the ZIKV outbreak and provides baseline data for longitudinal studies on ZIKV pathogenesis.

**IMPORTANCE** The Latin American Zika virus (ZIKV) outbreak had a major impact on reproductive health worldwide. The reasons for the massively increased reports of neonatal microcephaly in northeastern Brazil are still unclear. Beyond the technical limitations of laboratory diagnostics, unambiguous diagnosis of ZIKV as the cause of congenital malformations is hampered by similar clinical pictures elicited by other pathogens known as TORCH pathogens. We performed a case-control study comparing mothers of children with congenital malformations to age-matched controls from Salvador, Brazil, one of the areas most extensively affected by the ZIKV outbreak. The ZIKV and Chikungunya virus seroprevalence rates differed significantly, whereas the levels of maternal exposure to TORCH pathogens were similar between cases and controls. Our data support a link between maternal ZIKV infection and congenital malformations and suggest the occurrence of predominantly vector-borne ZIKV transmission in these cases. In addition, some highly prevalent TORCH pathogens may be misinterpreted as representative of ongoing ZIKV activity in the absence of exhaustive diagnostics in northeastern Brazil.

## Abstract (precis)

The Latin American Zika virus (ZIKV) outbreak had a major impact on reproductive health worldwide. The reasons for the massively increased reports of neonatal microcephaly in northeastern Brazil are still unclear. Beyond the technical limitations of laboratory diagnostics, unambiguous diagnosis of ZIKV as the cause of congenital malformations is hampered by similar clinical pictures elicited by other pathogens known as TORCH pathogens. We performed a case-control study comparing mothers of children with congenital malformations to age-matched controls from Salvador, Brazil, one of the areas most extensively affected by the ZIKV outbreak. The ZIKV and Chikungunya virus seroprevalence rates differed significantly, whereas the levels of maternal exposure to TORCH pathogens were similar between cases and controls. Our data support a link between maternal ZIKV infection and congenital malformations and suggest the occurrence of predominantly vector-borne ZIKV transmission in these cases. In addition, some highly prevalent TORCH pathogens may be misinterpreted as representative of ongoing ZIKV activity in the absence of exhaustive diagnostics in northeastern Brazil.

## OBSERVATION

During the 2015–2016 Zika virus (ZIKV) outbreak, a 20-fold increase in the incidence of neonatal microcephaly was observed after the large first epidemic wave in northeastern Brazil (1–3). In addition to microcephaly, ZIKV causes other fetal abnormalities summarized as congenital Zika syndrome (CZS), including skull and brain deformities, ocular abnormalities, arthrogryposis, and spasticity (4).

Despite the experimental evidence supporting ZIKV neuropathogenicity (5, 6), proving the etiologic role of ZIKV in neonates with neurological malformations is challenging. Laboratory diagnosis of ZIKV is hampered by the low sensitivity and specificity of virological tests (7). In addition, teratogenic substances and genetic disorders (8), as well as several pathogens other than ZIKV, can cause similar clinical presentations in fetuses and neonates (9, 10). These pathogens, grouped under the acronym TORCH, include, among others, Toxoplasma gondii, Listeria monocytogenes, Treponema pallidum, rubella virus (RUBV), cytomegalovirus (CMV), herpes simplex virus-1 (HSV-1) and HSV-2, varicella-zoster virus (VZV), and parvovirus B19 (PV-B19) (9).

In Brazil, routine antenatal screening is mainly performed for T. pallidum and T. gondii. In cases of suspected congenital malformations, additional laboratory testing is performed but usually does not include all TORCH pathogens. Additionally, usage of different methods hinders comparisons of laboratory results for these pathogens across sites. Information on TORCH pathogens and their potential association with suspected cases of ZIKV-associated congenital malformations is thus scarce. In Brazil, a case-control study performed in Recife reported no statistically significant difference in the levels of maternal exposure to RUBV, CMV, and T. gondii in 32 cases of suspected CZS and 64 controls (11). Additionally, cohort studies in the Caribbean and Rio de Janeiro, Brazil, failed to observe fetal abnormalities in 11 combined cases of ZIKV coinfections with HIV-1, CMV, T. gondii, or T. pallidum (12, 13). In contrast, a cohort study performed in São Paulo, Brazil, reported one case of ZIKV coinfection with T. gondii with abnormal neuroimaging findings (14). TORCH pathogens may be overlooked as causes of congenital malformations attributed to ZIKV during the outbreak or unrecognized cofactors of suspected CZS or may impact clinical presentations in cases of suspected CZS in northeastern Brazil.

To investigate exposure to the emerging arboviruses ZIKV and CHIKV in comparison to exposure to established TORCH pathogens, we conducted exhaustive serological investigations in 32 mothers of children born with congenital malformations (termed cases) and 160 mothers of children born without congenital malformations (termed controls) from a highly ZIKV-affected region in northeastern Brazil (1). For every case, 5 controls were matched by age (±2.0 years). The resulting age distributions of cases (mean, 26.8 years; standard deviation [SD], 6.6) and controls (mean, 28.9 years; SD, 6.6) did not differ significantly (*t* test, *P* = 0.09). Beyond age, data on education and ethnicity were available for around 70% of cases and controls. The study comprised mainly mixed-race individuals (80.0% of cases and 92.2% of controls; χ^2^, *P* = 0.066) with completed secondary schooling (85.7% of cases and 92.2% of controls; Fisher’s exact test; *P* = 0.402).

All cases showed abnormal but heterogeneous clinical and neuroimaging findings, preventing unequivocal diagnosis of suspected CZS based on clinical presentation (see Table S1 in the supplemental material). Common findings included microcephaly (81.3%; 26/32) followed by intracranial calcifications (75.0%; 24/32), ventriculomegaly (56.3%; 18/32), dysgenesis of the *corpus callosum* (28.1%; 9/32), and Dandy-Walker-like malformations (18.8%; 6/32). Hydranencephaly, porencephaly, and hydrocephalus (66.6%; 4/6), severe intracranial calcifications (66.6%; 4/6), and reduction of encephalic mass (50.0%; 3/6) were reported from 6 cases classified as suspected CZS despite normal head circumference at birth, consistent with a recent case series from the Caribbean (15).

10.1128/mSphere.00278-18.1 TABLE S1  Characteristics of suspected congenital Zika syndrome in cases. Download TABLE S1, PDF file, 0.1 MB. Copyright © 2018 Moreira-Soto et al. 2018 Moreira-Soto et al. This content is distributed under the terms of the Creative Commons Attribution 4.0 International license.

**FIG 1:**
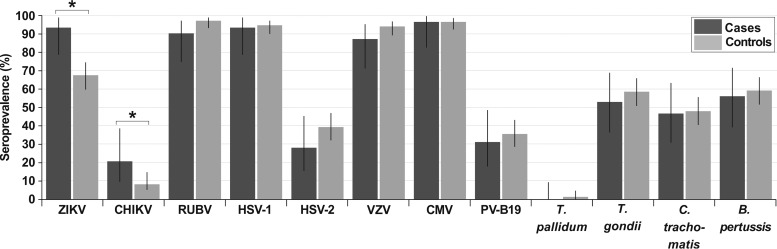
Seroprevalence rates of cases and controls. Seroprevalence rates are shown with adjusted Wald confidence intervals (column lines). Asterisks denote a *P* value of <0.05. Fisher’s exact tests were done when any cell count was below 5; otherwise, χ^2^ tests were done to compare seroprevalence rates.

The difference in ZIKV seroprevalence rates between cases (93.8%) and controls (67.8%) was statistically significant (Fisher’s exact text, *P* = 0.002, power = 93.9%). A similar discrepancy was observed for CHIKV seroprevalence rates between cases (20.7%) and controls (8.2%; χ^2^, *P* = 0.039, power = 53.0%), suggesting higher exposure of cases to these vector-transmitted pathogens. Nonetheless, only ZIKV was significantly associated with congenital malformations in conditional logistic regression analyses (*P* = 0.030; odds ratio, 4.0 [95% confidence interval {CI}, 1.1 to 14.1]). High ZIKV seroprevalence in cases was unlikely to be explained by potential cross-reactive antibodies affecting ZIKV serological tests elicited by previous dengue virus (DENV) infections, because DENV seroprevalence was higher in controls (89.1%) than in cases (74.1%; χ^2^, *P* = 0.1). Seroprevalence of TORCH pathogens in cases and controls ranged from very high (87.5% to 97.5%) for HSV-1, RUBV, VZV, and CMV to moderate (28.1% to 59.4%) for Chlamydia trachomatis, Bordetella pertussis, T. gondii, PV-B19, and HSV-2 and low (0.0% to 1.3%) for T. pallidum, and no significant differences were found between groups (Fig. 1) (Table 1).

**TABLE 1:** Seroprevalence rates in cases and controls

Pathogen	Seroprevalence cases (%)	95% CI^a^	Seroprevalence controls (%)	95% CI	Odds ratio^b^	95% CI	*P^d^*
ZIKV	93.8	78.8–99.3	67.8	59.9–74.8	7.1^c^	1.6–31.1	**0.002**
CHIKV	20.7	9.5–38.8	8.2	5.2–14.8	2.9^c^	1.0–8.4	**0.039**
DENV	74.1	55.1–87.1	89.1	79.2–95.6	0.3	0.0–1.0	0.100
RUBV	90.6	75.0–97.5	97.5	93.5–99.2	0.2	0.0–1.2	0.092
HSV-1	93.8	78.8–99.3	95.0	90.3–97.6	0.8	0.1–3.9	0.673
HSV-2	28.1	15.4–45.5	47.1	32.1–47.1	0.6	0.3–1.4	0.230
VZV	87.5	71.3–95.6	94.4	89.5–97.2	0.4	0.1–1.5	0.236
CMV	96.9	82.9–100.0	96.9	92.7–99.0	1.0	0.1–8.9	1.000
PV-B19	31.3	17.8–48.7	35.6	28.6–43.3	0.8	0.4–1.8	0.635
C. trachomatis	46.9	30.9–63.6	48.1	40.5–55.8	0.9	0.4–2.0	0.897
B. pertussis	56.3	39.3–71.9	59.4	51.8–66.7	0.9	0.4–1.9	0.742
T. pallidum	0.0	0.0–9.3	1.3	0.0–4.7	0.9	0.0–20.8	1.000
T. gondii	53.1	36.5–69.1	58.7	51.0–66.1	0.8	0.4–1.7	0.556

a CI, confidence interval.

b Data represent results from bivariate comparisons.

c In conditional logistic regression analyses, odds ratios were as follows: ZIKV (*P* = 0.030; odds ratio, 4.0 [95% CI, 1.1 to 14.1]) and CHIKV (*P* = 0.084; odds ratio, 2.8 [95% CI, 0.1 to 9.2]).

d Data were calculated using χ^2^ tests and Fisher’s exact tests when any cell count was below 5. Bold type denotes statistical significance.

Seroprevalence rates from our study were consistent with previous studies showing high (87.8%) VZV seroprevalence (16), moderate (64.9%) *T. gondii* seroprevalence (17), and low (2.8%) *T. pallidum* seroprevalence (18) in northeastern Brazilian adult women, suggesting robustness of our results. In the Zika case-control study performed in Recife, northeastern Brazil, the seroprevalence of T. gondii in cases and controls was 44% to 53% and thus was not significantly different from the range seen in our study (χ^2^, *P* > 0.05) (11). However, the seroprevalence rates of CMV (88% to 76%) and RUBV (63% to 76%) were lower in both cases and controls from Recife than in our study (Table 1; χ^2^, *P* < 0.05) (11). These differences might be explained by variations in study designs, regional differences, and differential performance of the diagnostic assays used in routine antenatal screening compared to our targeted serological investigation. Of note, since the Brazilian health system performs routine vaccination for VZV, RUBV, and B. pertussis, we cannot differentiate immune responses elicited by vaccination from those elicited by infection with wild-type pathogens (19).

The factors underlying the increase in potentially ZIKV-associated microcephaly cases in northeastern Brazil remain unclear. Since Bahia and several other northeastern Brazilian states are among the poorest regions of Brazil, poverty may be a general and yet unspecific effect modifier of ZIKV-associated microcephaly (1). The similarities in ZIKV and CHIKV seroprevalence patterns suggest a relatively higher exposure of cases to the main mosquito vector, Aedes aegypti. This may be potentially associated with lower socioeconomic status, implying residence in more densely populated areas with less-regular garbage recollection, providing enhanced availability of virus for mosquito vectors and higher mosquito density. This hypothesis is consistent with area-level analyses of socioeconomic factors affecting ZIKV exposure in Bahia (1) and affecting occurrence of cases of microcephaly in Recife (20).

Sexual ZIKV transmission has been previously described (21) and may play an important role in ZIKV congenital pathogenesis. However, similar levels of exposure of cases and controls to sexually transmitted infections suggest predominantly vector-borne ZIKV transmission in our study population. Of note, our results cannot be extrapolated to other geographical settings, since prevalence rates of TORCH pathogens and ZIKV might differ considerably between regions. Studies comparing levels of ZIKV seroprevalence in groups of sexually active and non-sexually active populations and in different social strata would be desirable to assess the significance of sexual ZIKV transmission per site.

Our study was limited by lack of routine screening for genetic and environmental causes of congenital malformations and by the absence of samples longitudinally collected from mothers during pregnancy and from neonates after birth, which hindered definite distinction between lifetime exposure and acute maternal infection during pregnancy potentially leading to congenital infection of the fetus. The strengths of our study included the exhaustive screening performed in an age-matched cohort, usage of only one test for TORCH pathogens to minimize biases that could distort test accuracy and reproducibility (22), and usage of multiple serological methods to accurately detect arbovirus exposure.

Our data strongly support ZIKV infection as a cause of severe congenital malformations in northeastern Brazil. Our baseline data for TORCH pathogens will inform subsequent epidemiological studies investigating ZIKV pathogenesis and the apparent accumulation of congenital malformations observed in northeastern Brazil (23). High TORCH seroprevalence rates suggest frequent exposure to TORCH pathogens in northeastern Brazil that must not be interpreted as evidence for ongoing ZIKV activity in the region without exhaustive diagnostics.

### Study population.

The nested case-control study encompassed 32 mothers of children born with congenital malformations and 160 mothers of children born without congenital malformations attending the University of Bahia Climério de Oliveira maternity ward. The study was approved by the Institutional Research Ethics Board under protocol no. 1.408.49. Cases and controls were sampled at the time of delivery during the same time period between May 2015 and October 2016. All patients who were attended to during the study period accepted participation in the protocol. Microcephaly cases were identified when the measurement of the cephalic circumference was 2 standard deviations below that of the corresponding gestational age, based on intergrowth charts from the World Health Organization in addition to clinical and imaging data (Table S1) (24).

### Serology.

Commercially available IgG enzyme-linked immunosorbent assays (ELISAs) for ZIKV and CHIKV were used according to the manufacturer’s instructions (Euroimmun, Lübeck, Germany). Due to potential cross-reactivity of ZIKV- and DENV-specific antibodies in serological assays (1), 50% plaque-reduction neutralization tests (PRNT_50_) were done for ZIKV as described previously (7). Only study participants who yielded positive ZIKV test results in both ELISA and PRNT_50_ were considered ZIKV positive. To test for exposure to the endemic DENV, we performed an in-house competitive ELISA that uses a mutant envelope antigen of DENV designed to be robust against cross-reactivity with ZIKV-specific antibodies, as previously described (25). Due to insufficient sample volumes, only 27 cases and 135 corresponding controls matched 1:5 were tested against DENV. Testing for TORCH pathogens was done using a commercially available immunoblot test (Euroline [anti-TO.R.C.H.-10 profile]; Euroimmun) which detects IgG antibodies against T. gondii, RUBV, CMV, HSV-1, HSV-2, VZV, PV-B19, and T. pallidum (the classical TORCH pathogens) as well as Bordetella pertussis and Chlamydia trachomatis (which cause severe disease in neonates). Automated readout of scanned immunoblots was performed using EUROLineScan software (Euroimmun).

### Statistical analyses.

Bivariate analyses were done using GraphPad Prism 5 (GraphPad Software, Inc., La Jolla, CA, USA) and conditional logistic regression with forward exclusion of variables in SPSS V23 (IBM, Ehningen, Germany) using the variables with *P* values of <0.05 in bivariate analyses, and power was calculated using OpenEpi 3.01. Statistics were calculated with 95% confidence intervals.
