# Supplementary material for: Exhaustive TORCH Pathogen Diagnostics Corroborate Zika Virus Etiology of Congenital Malformations in Northeastern Brazil
Source: mSphere. 2018 Aug 8;3(4):e00278-18. doi: 10.1128/mSphere.00278-18 (PMC6083096; doi:10.1128/mSphere.00278-18)
Supplement: TABLE S1 [file sph004182616st1.pdf]

Supplemental Table S1

ID	Neonatal sex	Gestational age (weeks)	Cephalic perimeter (cm)	Weight (gr)	Height (cm)	US/CT observations	APGAR
**ZK005**	F	38	28.0	2622	44.5	Intracranial calcifications, reduced encephalic mass, gross parenchymal and periventricular calcification, ventriculomegaly, agenesis of the *corpus callosum*	9/10
**ZK006**	F	39	28.0	3024	45.3	Dysgenesis of the *corpus callosum*, cerebral atrophy	8/9
**ZK011**	F	37	27.5	1840	39.5	Oligohydramnios, arthrogryposis, reduced encephalic mass, gross parenchymal and periventricular calcification, ventriculomegaly, dysgenesis of the *corpus callosum*	9/9
**ZK012**	F	38	27.0	2520	43.5	Oligohydramnios, arthrogryposis. Gross periventricular calcifications in basal nucleus, ventriculomegaly, severe microcephaly	8/9
**ZK016**	F	40	30.5	2774	45	Parenchymal and periventricular calcifications, intracranial haemorrhage, bone fractures	7/9
**ZK018**	M	40	27.0	2900	45	Gross periventricular calcifications in basal nucleus, ventriculomegaly	7/9
**ZK019**	M	36	26.5	2035	47	Brachycephaly, supra-cerebellar lesion suggesting porencephaly, hypotelorism, hypoplasia of nasal bone, cardiomegaly with hypertrophy of the ventricular walls	nt
**ZK024**	M	41	30.0	3466	48	Large ventriculomegaly, calcifications and destructive lesions on cerebellum, *ostium secundum* atrial septal defect	6/7
**ZK025**	F	41	31	1906	47	Large ventriculomegaly with calcifications and destructive lesion on cerebellum, hypoplasia of nasal bone, narrow thorax, pulmonary hypoplasia	2/2
**ZK027**	M	40	30.0	3088	50	Discrete ventriculomegaly, extensive periventricular calcifications, cisterna magna enlargement and destructive lesions in the posterior fossa, Dandy-Walker-like malformations	9/9
**ZK028**	M	39	28	2350	47	Ventriculomegaly, mega cisterna magna with agenesis of cerebellar vermis, periventricular calcifications, Dandy-Walker like malformations	2/3
**ZK033**	M	To term	Normal	3358	nt	Hydrocephalus, Cardiopathy and *situs inversus* within 6 months after birth	9/9
**ZK063**	F	40	37	3528	51	Porencephaly in cerebral parenchyma with cerebellar hypoplasia, Dandy-Walker like malformations within 6 months after birth	6/9
**ZK064**	F	39	27.5	2700	44	Severe ventriculomegaly, reduced cerebral parenchyma with periventricular calcifications, parenchyma reduction in both cerebral hemispheres, Dandy-Walker-like malformations	8/9

**ZK070**	F	37	32	2812	45	Intracranial punctiform calcifications in both cerebral hemispheres, hypodense parenchyma within 6 months after birth	7/9
**ZK072**	F	40	31.0	2974	48	Severe ventriculomegaly, reduced cerebral parenchyma with periventricular calcifications, parenchyma reduction in both cerebral hemispheres, hydranencephaly within 6 months after birth	8/9
**ZK290**	nt	nt	Nt	nt	nt	Microcephaly, 7 months abortion	nt
**ZK522**	F	40	27.5	2490	43	Parenchymal atrophy, periventricular and basal nuclei calcifications, ventriculomegaly, bilateral subependymal cysts, dysgenesis of *corpus callosum*	9/10
**MO16**	F	40.3	28.5	3110	47	Ventriculomegaly and periventricular calcifications, atrial septal defect	7/8
**MO18**	F	40	25	2100	41	Colpocephaly, cerebellar hypoplasia, enlargement of ventricles, reduced size of *corpus callosum*, olygohydroamnios	8/9
**MO19**	F	37	30	2744	42	Severe calcifications, ventriculomegaly, lissencephaly	9/9
**ZK739**	M	38	28	3024	47	Cortical atrophy, subcortical calcifications, ventriculomegaly, dysgenesis of the *corpus callosum*	9/9
**MO22**	M	32	28	2694	48	Atrophy of the cortex and base nucleus, cortical, paraventricular and base nucleus with calcifications, ventriculomegaly, dysgenesis of *corpus callosum*	9/9
**MO23**	M	42	31	3296	49	Several parenchymal calcifications, cortical atrophy, enlargement of the periencephalic space	7/9
**MO**27	M	To term	32	2730	43	Microcephaly, periventricular and basal nuclei calcifications, bilateral subependymal cysts, moderate ventriculomegaly with Dandy-Walker-like malformation	8/9
**MO35**	M	39	27,5	2502	45	Parenchymal and periventricular calcification, ventriculomegaly, cortical atrophy.	9/9
**MO213**	F	39	31	3636	nt	Diffuse parenchymal and ventricular calcifications, ventriculomegaly	9/9
**MO230**	M	40	29	3315	48	Dysgenesis of the *corpus callosum*	9/9
**ZK843**	M	40	29	2982	nt	Cortical atrophy, dysgenesis of the *corpus callosum*, parenchymal and periventricular calcifications	9/10
**ZK844**	F	38	30	2382	43	Dysgenesis of the *corpus callosum*, parenchymal atrophy with diffuse microcalcifications	9/9
**ZK852**	F	41	33	3370	46	Sulcus and gyrus reduction, cortical atrophy with *ex-vacuo* ventriculomegaly, diffuse calcifications, diminished periventricular white substance within 6 months after birth	nt
**ZK855**	M	37	36	2770	48	Hidranencephaly, Dandy-Walker-like malformation, arthrogryposis,	9/9

	gross encephalic and punctiform cerebellar calcifications within 6 months after birth

ID, patient study code; US, ultrasound; CT, computed tomography. Nt: not taken. The institutional research ethics board of the Federal University of Bahia Climério de Oliveira approved the study under protocol no. 1.408.49. All study participants signed informed consent before sampling at delivery. Microcephaly was diagnosed at birth when the head circumference was two standard deviations below that of the corresponding gestational age, based on intergrowth charts.
